# Effect of Artificial Food Additives on Lung Health—An Overview

**DOI:** 10.3390/medicina61040684

**Published:** 2025-04-08

**Authors:** Yousef Saad Aldabayan

**Affiliations:** Department of Respiratory Care, King Faisal University, Al-Ahsa 31982, Saudi Arabia; yaldabayan@kfu.edu.sa

**Keywords:** preservatives, synthetic colorants, respiratory health, asthma, chronic obstructive pulmonary disease (COPD), ultra-processed foods, inflammation

## Abstract

This review focuses on the potential health risks of artificial food additives, especially their effects on lung health. Preservatives, synthetic colorants, and flavor enhancers, which are commonly used in processed foods, play roles in worsening respiratory diseases, such as asthma and chronic obstructive pulmonary disease (COPD). These additives cause oxidative stress, systemic inflammation, and immune dysregulation, often through the gut-lung axis. The preservatives sodium nitrite and sulfites have the risk of causing bronchial hyper-responsiveness and allergic reactions. The synthetic colorant, Ponceau 4R, is also related to immune-mediated lung inflammation. Flavoring agents such as diacetyl contribute to occupational respiratory diseases like bronchiolitis obliterans. In animal models, prenatal exposure to additives, such as titanium dioxide (E171), might disrupt the development of respiratory neural networks, with long-term consequences. Ultra-processed foods (UPFs), which also contain a high concentration of additives, lead to systemic inflammation and impair lung function. Despite their wide usage, the use of these additives has become a warning sign due to their safety issue, particularly in sensitive people like children, pregnant women, and patients with pre-existing respiratory and chronic conditions. The review highlights the serious need for strict regulation and further research on the long-term effects of food additives on respiratory health. Policymakers should ban these food additives that are more harmful to human health. As an alternative to artificial additives, natural flavors and colors from fruits and vegetables, safe preservatives, and minimally processed ingredients can be used.

## 1. Introduction

Food can be defined as anything that offers nutritional value to any organism. The food may either be produced by plants or animals; it covers the essential nutrients that include carbohydrates, fats, proteins, and vitamins [1]. According to the federal Food, Drug, and Cosmetic Act, a food additive is any material that is not in itself a food but is intended for use to such an extent that it could, either directly or indirectly, become a component of food or otherwise affect the characteristics of food. It thus covers substances used in the manufacture, processing, preparation, treatment, packaging, or repacking of food articles or their transportation or storage. It also covers all sources of radiation intended for such purposes [2]. Food additives are comprised of organic and inorganic materials, which include enzymes, and they are applied at low concentrations to achieve some objectives. Such objectives include preservation of nutrients, extension of the shelf life of food products, and sensory attributes, which include texture, consistency, taste, flavor, and color [3].

Additives can be broadly classified into six main categories, namely preservatives, nutritional additives, flavoring agents, coloring agents, texturizing agents, and miscellaneous additives. Many additives have several functions in food products. Additives can be grouped in various ways depending on the origin, source, or functional activity of the additives in food. Many food additives come from both natural sources and synthetic processes. The natural colorants annatto is derived from the seeds of the achiote tree and is used for coloring snacks and cheese; curcumin, a compound in turmeric, is used in various food dishes; agave syrup is a sweetener produced from the agave plant. Synthetic colorants are produced in the laboratories [4].

Traditionally, these food additives can be categorized as coming from natural foods, such as spices, herbs, roots, and essential oils. Soybeans and corn contribute to stabilizing food products, while beet powder extracted from beets is a colorant, although caramel, manufactured through the caramelization of sugar, is similarly used as a colorant [4].

Synthetic food additives, on the other hand, are produced through chemical synthesis, that is, one or more chemical compounds. Some of the synthetic additives include aspartame, which is a preservative based on the aspartic acid compound C_4_H_5_O_4_NH_2_; erythrosine, which is the disodium salt of 2,4,5,7-tetraiodofluorescein and a coloring agent; and tartrazine, a trisodium compound [(4E)-5-oxo-1-(4-sulfonatophenyl)-4-[(4-sulfonatophenyl) hydrazono]-3-pyrazolecarboxylate], and this compound is also a coloring agent [4].

Direct food additives are intentionally added for specific purposes, whereas indirect food additives are typically unintended substances that may come into contact with food during various stages of its lifecycle (production, storage, packaging, and transportation) Indirect additives, such as erythrosine (red), canthaxanthin (orange), and annatto bixin (yellow-orange), are added to food to make it more attractive to consumers without providing any nutritional value. Small amounts of packaging materials may leach into food while in storage. Food preservatives are a class of additives that prevent spoilage by inhibiting the growth of pathogenic microorganisms, such as Clostridium spp., Bacillus cereus, and Staphylococcus aureus. Thus, they ensure food safety and shelf stability [5].

## 2. Classifications of Food Additives [5]

There are many classes of food additives, but many additives find applications in more than one class.

Antimicrobial Agents: These agents retard the growth of microorganisms so that food cannot spoil. Some examples of such preservatives are traditional types like vinegar and salt, calcium propionate, and sorbic acid. Such additives are commonly found in baked goods, salad dressings, cheeses, margarine, and pickled foods.

Antioxidants: These are the substances added to fats and foods containing fats to delay oxidation, which in turn can preserve quality, flavor, and shelf life. Ideally, antioxidants must not add bad odor, flavor, or color to the food. They must act at very low concentrations. Most importantly, they must be fat-soluble and nontoxic. Common synthetic antioxidants are butylated hydroxyanisole (BHA), butylated hydroxytoluene (BHT), propyl gallate (PG), and tertiary butylhydroquinone (TBHQ), all of which are phenolic compounds. Other antioxidants like thiodipropionic acid and dilauryl thiodipropionate also find their application in food preservation.

Coloring Agents: They are stabilizers and fixatives, as well as retention agents, and they can be either synthetic or natural. Though most color additives cannot perform a nutrient function, they remain essential for the aesthetics of foods because consumers associate certain flavors with specific colors or consider the foods fresh because of coloration. Colors are usually added to replace the ones that are lost during processing or to meet consumers’ demand for a natural appearance. Natural food colors extract color from sources such as seeds, flowers, insects, and foods. For instance, bixin, a well-known red pigment extracted from the seed coat of *Bixa orellana*, or the lipstick pod plant, is not carcinogenic. Annatto, which comes from the pulp of *Bixa orellana* seeds, serves as a yellow to red colorant in butter, cheese, margarine, and other consumable products. Other examples are carotene from carrots for margarine, saffron for flavoring and coloring curries and salad dressings, and cochineal, a natural red derived from the insect *Coccus cacti.* Other colors include grape skin extract and caramel, the brown color produced from burnt sugar. There are also some synthetic food colorants, such as erythrosine and carmoisine. These preservatives attempt to make food look more attractive and have a color associated with the perceived taste (such as red for cherry or green for lime).

Bleaching Agents: Peroxides bleach foods such as wheat flour and cheese to give them a more attractive appearance.

Chelating Agents: Chelating agents, although not antioxidants, prevent oxidation by binding metals that catalyze the process. They are applied primarily to counter food discoloration, flavor change, and rancidity. In addition, an amount of citric acid to the extent of 0.1–0.3% along with an antioxidant at 100–200 ppm is applied. EDTA stands for ethylenediaminetetraacetic acid. It is a chelating agent allowed most often, and its application as a food preservative is also permitted by the United States Food and Drug Administration in terms of calcium disodium EDTA and disodium EDTA. Other chelating agents are malic acid and tartaric acid.

Nutrient Supplements: Supplements of nutrients are added to foodstuffs either to replace those that are lost in processing or storage or to make them more nutrient-rich than when processed. When processed, there may be a reduction of some nutrients in the foods. Additives replace these lost nutrients to regain the original concentration. For instance, during the milling of white flour, the brown outer layer of the wheat grain is stripped off as it is abundant in vitamins and minerals. Instead of the removal of the brown layer, thiamine, nicotinic acid, iron, and calcium are replaced in the flour. Vitamin C is sometimes supplemented with canned citrus fruits to replace the vitamin removed by processing.

Acids: Food acids are applied to make foods more acidic in flavor, apart from acting as preservatives and antioxidants. The most common food acids are vinegar, citric acid, tartaric acid, malic acid, fumaric acid, and lactic acid.

Preservatives: Preservatives are chemicals that inhibit or retard the growth of microorganisms to delay the deterioration of foods and prevent spoilage. About 20% of the world’s food supply is lost to microbial spoilage. Preservatives work by disrupting microorganisms on a cell membrane, enzymatic, and even genetic level. Various preservatives may either be chemically synthesized or obtained through elements such as sugar, salt, and acids. As the shelf life of food increases with preservatives, flavor change, color loss, and oxidation of oils are largely prevented. They also preserve foods, which may include cured meats, from harmful toxins such as botulinum, which can cause food poisoning [5].

pH Control Agents: Additives that make up acids, alkalis, and buffers to control the pH of foods. Acidity or alkalinity will be adjusted to influence food properties, which include their flavor, texture, and quality during cooking (examples: citric acid, acetic acid, phosphoric acid, and lactic acid).

Anticaking Agents: Anticaking agents (calcium silicate, sodium citrate, magnesium carbonate, etc.) prevent particle clumping in moist conditions, allowing such free-flowing powders as salt and milk powder to remain usable.

Leavening Agents: These agents, such as baking soda, yeast, and ammonium bicarbonate, make the final products light and airy. Although yeast is still a significant leavening agent in bread-baking, ammonium salts are often used to provide nitrogen for yeast growth. Salt phosphates, sodium, and calcium are used to control pH during baking.

Antifoaming Agents: These agents reduce or inhibit the formation of foam in foods. They improve the texture and processing efficiency of foods (examples: vegetable oils, polyglyceral esters, and fatty acids).

Bulking Agents: Additives like starch, dextrin, oligo-fructose, etc., act as bulking agents, increasing the volume of food without altering its nutritional value.

Color Retention Agents: Unlike coloring agents, these substances (vitamin C, vitamin E, and citric acid) are used to preserve a food’s natural color rather than add new colors.

Emulsifiers: Emulsifiers assist in suspending liquids that, by their nature, separate from each other. This includes oil and water. Emulsifiers are mainly used in dairy, confectionery, margarine, salad dressings, and shortenings for uniformity. Moreover, emulsifiers allow suspensions of water and oil in products such as mayonnaise, ice cream, and homogenized milk. Peanut butter can contain up to 10% of emulsifiers, which gives it a smooth texture. Monoglycerides, diglycerides, lecithin, and polysorbates are the best examples of emulsifiers.

Flavors and Flavor Enhancers: Flavoring additives are those substances, which may be natural or synthetic, that are added to food for characteristic flavor. The flavor enhancers are ingredients that do not produce flavor themselves but synergistically enhance the flavors of others. Flavors and flavor enhancers constitute the largest category of food additives. Natural flavoring materials include spices, herbs, roots, and essential oils, which were traditionally used as flavorings. However, the availability of natural flavorings is limited, and the concentrations of active flavor components in such materials are often minuscule. To illustrate, one ton of a certain spice could be needed to yield just 1 g of the flavoring compounds. As a result, natural flavorings are quickly being substituted with synthetic flavoring compounds because these are relatively easy to synthesize in large volumes. The flavoring compounds largely are esters, aldehydes, ketones, alcohols, and ethers. They are primarily prepared and commonly replace the natural alternatives. Some illustrations of synthetic flavoring additives are amyl acetate for bananas, methyl anthranilate for grapes, and ethyl butyrate for pineapple. Most artificially flavored foods consist of thousands of individual compounds. For instance, imitation cherry flavor may contain up to fifteen esters, alcohols, and aldehydes.

MSG is a flavor enhancer derived from the sodium salt of the naturally occurring amino acid glutamic acid. One of the most used and well-known flavor enhancers is MSG, which is used extensively in foods, particularly in meats, fish, poultry, vegetables, sauces, soups, and marinades to add flavor. When appropriately used, MSG is safe; however, it was once associated with reports of damage to the brains of young mice that had been injected with MSG.

Flour Improvers: Flour improvers are made up of bleaching and maturing agents, which are indispensable to the flour-milling and bread-baking industries. Freshly milled flour is yellowish and produces a weak dough, as a result of which poor-quality bread is obtained. The storage of the flour for several months before use improves its color and baking qualities. Chemical agents used as flour improvers are generally oxidizing agents and can be applied for bleaching, dough improvement, or both. For instance, benzoyl peroxide is only applied to bleach flour, while chlorine gas, chlorine dioxide, nitrosyl chloride, and nitrogen oxides are applied to bleach as well as to enhance the dough. Oxidizing agents include potassium bromate, potassium iodate, calcium iodate, and calcium peroxide only for dough-enhancing purposes.

Glazing Agents: Glazing agents are agents that impart a shiny appearance or a protective coating to foods, thereby improving their appearance and texture.

Humectants: Humectants are moisture-retention agents for food. They have a variety of functions, such as controlling viscosity and texture, bulking, preservation of moisture, reduction of water activity, regulation of crystallization, and retention of softness. They also help in the rehydration of dehydrated foods and solubilization of flavor compounds. Common humectants include polyhydroxy alcohols, which are water-soluble and hygroscopic, such as propylene glycol (CH_3_·CHOH·CH_2_OH), glycerol, sorbitol, and mannitol [CH_2_OH (CHOH)_4_CH_2_OH]. Except for propylene glycol, these sugar derivatives are mainly derived from nature.

Tracer Gas: Tracer gases (polyethylene glycol, shellac, beeswax, etc.) are used to test food packaging for its integrity, thus preventing food from reaching the atmosphere and thus helping preserve its shelf life.

Stabilizers and Thickeners: These additives enhance and stabilize foods’ textures, prevent crystallization, stabilize emulsions and foams, minimize the stickiness of icings, and help encapsulate flavors. Common stabilizers and thickeners are polysaccharides, such as gum Arabic, carrageenan, agar-agar, alginic acid, starch, carboxymethylcellulose, and pectin. Another commonly used one is gelatin, a substance that is not a carbohydrate. These agents are hydrophilic; they disperse in solutions as colloids, swell in hot and cold water, and thereby thicken the food. Common foods that include stabilizers and thickeners are gravies, pie fillings, cake toppings, chocolate milk drinks, jellies, puddings, and salad dressings. These additives add viscosity without appreciably altering other properties.

Sweeteners: Sweeteners add flavor to food and often substitute for sugar. These additives minimize the calorie count of food. They are typically recommended to patients who have diabetes, who care about their dental health, or those suffering from diarrhea because they avoid a surge in blood sugar.

Preservatives: Preservatives are additives that prevent the spoilage of meat, improve its color and flavor, inhibit microbial growth, and prevent toxin formation. Sodium nitrite is a preservative that has been in use for a long time to maintain the color and safety of meat products. When added to meat, nitrite is converted to nitric oxide, which binds with myoglobin to form a heat-stable pigment called nitrosyl myoglobin [5]. This process also enhances flavor, inhibits the growth of pathogenic bacteria like Clostridium and Streptococcus, and reduces the temperature needed to kill *Clostridium botulinum.*

Other Additives: Numerous food additives have functions other than those described above. Clarifying agents, including bentonite, gelatins, and synthetic resins, are used to remove haze, sediments, and oxidation products in fruit juices, beers, and wines. There are food enzymes that, once added, affect specific changes to food. Such enzymes include renin for cheese making, papain for tenderizing meat, and pectinase for the clarification of beverages. Fruits and vegetables become crunchy by firming agents, which comprise aluminum sulfate and calcium salts. Freezing agents, such as liquid nitrogen and dichlorofluoromethane, rapidly chill foods by evaporating at room temperature. Solvents like alcohol, propylene glycol, and glycerol dissolve flavor, color, and other ingredients. In instant food packets, packaging gases, such as inert gases like helium and neon, are used to prevent oxidation and other changes.

Food additives like emulsifiers, sweeteners, colorants, and microparticles or nanoparticles are added to UPFs very often [6].

## 3. Adverse Effects of Additives on Human Health

Food additives can have a wide range of effects, from immediate reactions to serious long-term effects, especially if a person is exposed repeatedly or if these substances accumulate in the body. Short-term effects might include fluctuations in energy levels, headaches, or changes in behavior and immune system responses. Long-term exposure has been associated with an increased risk of major health conditions, such as cardiovascular diseases and cancer. Others, even the newer chemical preservatives, also cause a scare since they might lead to respiratory complications among other health complications [5].

Immune System. Additives might also have an impact on the immune system whereby, in some cases, this can cause severe anaphylactic shock and therefore necessitate emergency medical services. Generally, most of the harmful additives arise from two principal sources. The first is deliberately added to foods during processing. These include preservatives, flavor enhancers, colors, sugars, and texture agents. Their presence can easily be determined from the label since they are normally declared on such labels. The second source comprises additives that come into contact with food in its packaging, storage, or handling. Most of these additives are not declared on the label. It is particularly essential to avoid food additives in children’s products since most commercially processed foods with preservatives contribute to several health issues in the long run [5].

### 3.1. Malnutrition and Associated Dangers of Food Additives

Food additives can also negatively affect the nutritional content of foods, which may lead to unhealthy diets and unknown malnutrition. Most processed foods with high salt, sugar, and fat levels are usually accompanied by additives. For instance, sucrose contributes calories without providing any essential nutrients, whereas fats, although nutrient-rich, are calorie-dense and often lacking in necessary vitamins or minerals. Processing methods often remove natural nutrients from foods, and although some vitamins and minerals may be added back, the nutritional value remains insufficient compared to the calorie content. These foods, therefore, lead to high-calorie but low-nutrient diets, which may lead to suboptimal nutrition and marginal malnutrition.

Food additives are necessary for preserving food and extending shelf life. However, they may also lead to health concerns. Some people may be sensitive to certain additives, leading to conditions such as asthma, hyperactivity, attention deficit disorders, hay fever, eczema flare-ups, skin rashes, headaches, chest tightness, or vomiting [1].

### 3.2. Risks of Specific Additives and Preservatives

Some commonly used food additives and preservatives have been linked to health risks, including the following [1]:

Tartrazine: A synthetic yellow coloring agent associated with genotoxicity when consumed in excessive amounts over long periods.

Boric Acid: Used as a preservative in some dairy and meat products, it can be harmful when consumed in large amounts, though its risks are not widely known.

Nitrites and Nitrates: Added to processed meats, these preservatives can combine with hemoglobin, thus raising the risk of cancers like brain tumors and leukemia, especially in children.

BHA and BHT: These preservatives may cause hyperactivity in children, damage to the lungs, liver, and kidneys, and are suspected carcinogens at high doses.

Artificial Sweeteners (e.g., Saccharin, Aspartame, Sucralose): Associated with side effects like headaches, breathing problems, skin allergies, gastrointestinal issues, and migraines. Sucralose contains a chlorine-based compound that is carcinogenic, while aspartame has been connected to depression and mood disorders.

These examples highlight the importance of strict regulation of food additives and the need for greater awareness of their long-term health implications [5].

### 3.3. Further Dangers of Food Additives

Other risks from food additives are as follows [7]:

Benzoates: These induce allergic reactions in the form of skin rashes and asthma. They are suspected to cause damage to the brain.

Bromates: These are known to lower the nutrient value of food, and they are also known to cause nausea and diarrhea.

Butylates: These are related to higher cholesterol levels and liver or kidney damage.

Caffeine: Widely used as a flavor enhancer and coloring agent, too much caffeine can cause nervousness, erratic heartbeats, and even heart defects in extreme cases.

Saccharin: Has been known to cause toxic effects on the skin, digestive tract, and heart. It has also been associated with causing bladder cancer.

Red Dye 40: Cancers and birth defects have been associated with this artificial color.

Mono- and Di-Glycerides: They have been associated with causing birth defects, genetic mutations, and cancers.

Caramel Coloring: Added for flavoring and color, caramel causes vitamin B6 deficiency and mutations in DNA, possibly linked to cancers.

Sodium Chloride, Also Known as Salt: Intake of high levels of salt is associated with conditions such as high blood pressure, kidney failure, strokes, and heart attacks.

Food additives play a very important role in modern food production, although their health impacts cannot be ignored. Regulating these substances and raising public awareness can greatly minimize the risks associated with consumption.

## 4. Effect of Artificial Food Additives on Lungs and Respiratory Health

**Figure 1:**
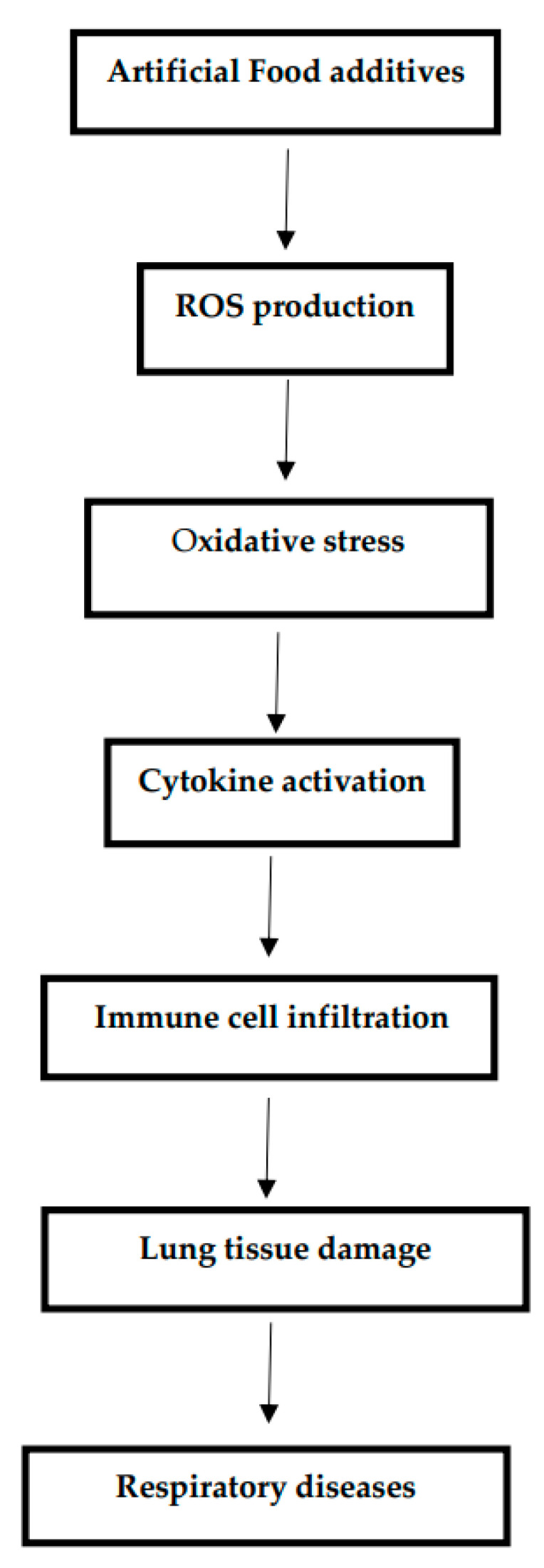
Mechanisms of Artificial Food Additives in Lung Inflammation and Oxidative Stress.

There is, however, limited research on how food additives made synthetically affect the lungs. Other studies have reported possible links between substances and respiratory disorders. In this article, the adverse effects of artificial food additives on the lungs and the underlying mechanism have been discussed [Table 1 and Figure 1].

### 4.1. The Influence of Food Preservatives, Coloring Agents, and Antioxidants on Allergic Diseases and Asthma

Zaknun et al. [8] reviewed the impact of food preservatives and colorants on respiratory disorders such as asthma. According to their results, preservatives such as sodium benzoate may exacerbate asthma symptoms and cause allergic reactions. These compounds can interfere with immune pathways and disturb the Th1/Th2 balance, which is essential in the regulation of allergic conditions. It has been widely documented that a greater intake of preservatives in a modern diet was associated with growing asthma and allergic diseases in industrialized societies.

The review also addressed the contribution of artificial food colorants to respiratory health. Some studies indicate that these additives could induce allergic reactions, which then present as asthma symptoms. The chemical structure of the additives can alter the immune response, and there is a propensity for a Th2-dominant immune profile associated with allergic reactions.

Both preservatives and artificial colorants are thought to affect the immune system in a manner that supports Th2-dominated responses. The immune shift leads to enhanced production of IgE, which plays a central role in allergic conditions such as asthma.

### 4.2. The Impact of Ultra-Processed Food and Their Additive Consumption on Asthma in Western Diets

According to the research carried out by Frontela-Saseta et al. [9], UPFs and their additives, including preservatives and colorants, play a significant role in the pathogenesis of asthma and other respiratory conditions. UPFs are nutritionally poor foods commonly consumed in Western diets and are rich in saturated fats, refined sugars, sodium, and artificial ingredients. These elements combine to produce systemic inflammation and oxidative stress that negatively affect the respiratory system.

Some key findings from the study include:

Preservatives and Colorants: These additives in UPFs may disturb the immune system. The gut-lung axis has been used to explain that the disruption caused is linked with diet-induced alterations in gut microbiota leading to inflammation and an imbalance in the immune system, worsening asthma and other respiratory conditions.

Systemic Inflammation: Systemic inflammation with UPFs has more to do with their formulation, not nutrient composition. Those chemical additives support chronic, low-grade inflammation, which increases the progressive rate of pulmonary decline and heightens asthma attacks.

Immune Dysregulation: Additives and contaminants, including bisphenols and phthalates from food packaging, further disrupt the immune balance. These chemicals are associated with a heightened risk of asthma and pulmonary disorders by affecting both the innate and adaptive immune systems.

The gut-lung axis: It could be harmful if UPFs continue to fuel the dysbiosis, meaning a reduction in the desired gut flora accompanied by a high increase in inflammatory by-products, which could thereby affect the overall lung function based directly on what has happened to gut health.

The study focuses on low UPF and stresses the call for further investigation into how these food additives affect one’s respiratory system. Intake of less processed food with whole substances is essential, thus improving health status and living.

### 4.3. Remodeling of the Lung Alveolar Structure in Response to a Combination of Food Additives

Shevchenko et al. [10] have studied the effect of two common food preservatives, sodium glutamate and sodium nitrite, on lung health. These compounds were found to induce severe morphometric alterations in the alveolar structure of rat lungs, resulting in thickened walls of the alveolus and changed form and number of alveolar cells. Indeed, sodium nitrite leads to interstitial and alveolar edema, which can contribute to allergic reactions and exacerbate respiratory illnesses such as asthma.

It was observed that Ponceau 4R, an artificial food coloring, is the cause of a respiratory problem; it can evoke hypersensitivity with symptoms like bronchitis and asthma. All these hypersensitivities were caused when proteins in the organism bound with such colorants, forming an allergenic complex, thus starting an immune reaction that resulted in lung damage.

The study found that preservatives and colorants both enhance lung inflammation. Increased alveolar macrophages and leukocyte infiltration were found to be increased in the study, which are signs of an inflammatory response that worsens asthma and other pulmonary conditions. These additives also were found to compromise the structural integrity of lung tissues, causing dystrophic changes in alveolar cells and decreased respiratory function.

The current results indicate a clear risk in using artificial food additives, particularly preservatives and colorants, towards the respiratory system. Such induction of inflammation as well as altering the morphology of lung tissue presents a problem with long-term use, particularly to those individuals who suffer from asthma or any form of lung disorder. More studies must be carried out to ascertain more on how the mechanisms influence the lung, then form diet guidelines on preventing the effects on vulnerable people.

### 4.4. The Impact of Sulfites in Food and Beverages on Asthma in Adults and Children

Article Gupta et al. [11] explored the role and possible side effects of food preservatives, additives, and colorants. They are the ones used mostly for enhancing the flavor, texture, and time before foods spoil, which mostly applies to processed and ready-to-eat foods, whereby artificial flavors and enhancers are very common. More of these additives play a specific functional role, but most pose health hazards, varying from short-term reactions to long-term risks after repeated exposure; in some cases, these risks may be fatal.

This article emphasizes sulfites, which include sulfur dioxide, and its salts, such as sodium bisulfite, as preservatives. Most often used preservative additives have been known to cause respiratory problems. Sensitivity to sulfites is a common finding in asthma, and the severity of bronchoconstriction may range from mild to fatal. Dried fruits and packaged snacks are some of the most common foods that contain sulfites; thus, intake of these products can trigger an asthma attack in sensitive individuals. It is estimated that 3% to 10% of asthmatic patients are sensitive to sulfites, with those who have poorly controlled asthma being more susceptible.

The article recognizes sulfites as a main culprit while noting that synthetic colors also might cause respiratory complications, specifically via allergic responses acting as asthma in patients, albeit with less prominence compared to the case of sulfites.

Sulfites cause respiratory problems by direct irritation of the airways and possible metabolic deficiencies in sulfite oxidase, an enzyme necessary for sulfite metabolism. In individuals with such deficiencies, sulfite accumulation can induce bronchospasms. New evidence also indicates that sulfites may interfere with gut microbiota, which could indirectly affect respiratory health through the gut-lung axis.

The findings underscore the need to pay attention to the health dangers posed by artificial food additives and, in particular, preservatives such as sulfites, in asthma sufferers and other respiratory compromised individuals. It suggests a preservative-free diet for children and asthma sufferers to achieve reduced danger and calls for further research on the relation of these additives with lung health and the establishment of dietary guidelines for sensitive groups.

### 4.5. A Brief Review of Flavoring-Related Lung Disease and New Mechanistic Insights

The study by Hubbs et al. [12] highlights the serious respiratory health hazards posed by artificial food additives, especially flavoring agents like diacetyl and its derivatives. These additives are widely used in the food processing industry to add flavor, but long-term occupational exposure has been associated with severe respiratory and pulmonary diseases. The study identifies conditions such as bronchiolitis obliterans, which is a lung disease causing irreversible airway obstruction, chronic bronchitis, and asthma, as potential consequences of long-term exposure to these substances.

The research highlights that flavoring agents like diacetyl can severely damage the respiratory system. They contribute to airway epithelial necrosis, fibrosis, and lung inflammation, which may lead to long-term problems like reduced lung function and chronic respiratory diseases. Diacetyl, in particular, is strongly associated with “popcorn lung”, a condition characterized by scarring of the lung tissue, often seen in workers exposed to high concentrations of this compound in food manufacturing environments.

Although the major health risks are quite pronounced in the case of food processing workers, the research concludes that the risk also exists for consumers because they are likely to be exposed to trace levels of such additives in processed foods. The research findings highlight the importance of tighter regulations and control measures aimed at reducing the risks related to flavoring agents like diacetyl and other artificial additives.

The study recommends enhancing awareness and regulation in the food industry, among other things: limiting the use of harmful flavoring agents in foods and implementing measures to protect the workers from occupational exposure.

### 4.6. Investigating the Link Between Ultra-Processed Foods and COPD: A Case-Control Study

The study by Salehi et al. [13] examined the effects of preservatives and colorants, which are typical constituents of UPFs, on respiratory and pulmonary health, including asthma and COPD. Although the study did not find a statistically significant association between UPF consumption and COPD risk, it identified several mechanisms through which these food additives may harm respiratory health.

The additives included in UPFs, such as preservatives and colorants, are linked with systemic inflammation and oxidative stress, which led to disruption of the immune function and contributed towards the development of asthma and COPD. Examples include preservatives like nitrites and synthetic antioxidants that can contribute to the development of reactive nitrogen species, with resultant oxidative stress and inflammation at the respiratory site. Furthermore, the high level of sodium in UPFs has been associated with airway inflammation and impaired lung function due to free sugars and saturated fats.

The study also cited other studies that have established that diets high in UPFs, which are typically low in nutritional value and high in artificial additives, cause systemic inflammation and oxidative stress. Such effects are dangerous to patients with pre-existing respiratory conditions because they can exacerbate symptoms such as bronchial hyperresponsiveness, chronic inflammation, and airway remodeling.

The research provides pathways through which these chemicals may bring about harm; however, further studies are warranted to clearly outline the direct and indirect effects of these additives on lung health. Subsequent research may therefore isolate the particular roles of preservatives and artificial colorants in the development and progression of respiratory diseases.

In summary, then, the study focuses more on investigating the biochemical and physiological effects of food additives on respiratory health as these impact dietary recommendations based on vulnerable populations.

### 4.7. Effect of Food Additives on Pulmonary Lymphoid Tissue Structure

Yeroshenko et al. [14] researched the impact of a mixture of food additives—monosodium glutamate, sodium nitrite, and Ponceau 4R—on the structure and function of pulmonary diffuse lymphoid tissue in rats. The authors concluded that these additives acted as antigens, provoking immune responses and antigen-driven differentiation of lymphoid cells. Morphological changes in the lymphoid tissue were observed throughout the experiment, including changes in the size, vascularization, and cellular composition.

The additives led to oscillating changes in the lymphoid tissue, with the initial response involving inflammation, while later, reorganization of tissues occurred. Reorganization decreased membrane thickness and lowered capillary numbers. With these structural defects and antigenic stimulation, the lungs underwent nonspecific inflammation. Sodium nitrite was related to hypoxia and oxidative stress, worsening the pulmonary damage further.

The outcomes of the study underline the harmful effects of food additives with preservatives and colorants on respiratory health: it reveals the potential effects of these substances in disrupting pulmonary immunity, inflammation, and structural changes in the lung tissue. This may indicate the role of food additives in the worsening or manifestation of respiratory and pulmonary disorders.

### 4.8. The Association Between Ultra-Processed Food Consumption and Chronic Respiratory Disease Mortality in Adults: Findings from a Prospective Cohort Study

In the study by Mekonnen et al. [15], UPFs and mortality from CRD have a highly explored relationship, focusing specifically on additives used by UPFs for preservatives and colorants. Strong evidence has been obtained whereby high consumption rates of UPFs are associated with a higher respiratory mortality rate like COPD. The highest UPF consumed group participants had a 10% higher risk of death related to CRD and a 26% higher risk of death related to COPD, compared with the lowest consumption group. The study also targeted additives and contaminants in UPFs, such as preservatives and synthetic colorants that induce oxidative stress, inflammation, and imbalance of the immune system. These mechanisms have been proven to exacerbate respiratory conditions by encouraging airway inflammation and compromising lung function. The chemical composition of UPFs, therefore, containing such additives, contributes to the progression and development of pulmonary disorders. It was also observed that the study had a dose-response relationship wherein even a 5% increase in UPF intake resulted in an increased mortality risk for CRD and COPD. The results indicate that dietary modification is relevant since small reductions in UPF intake led to improved results in respiratory health.

This research, therefore, puts emphasis on public health interventions and dietary changes as a means to reduce UPF consumption, particularly among at-risk populations. From the findings of this study, it is apparent that artificial additives play a paramount role in UPFs that adversely affect pulmonary health, and thus, reducing UPF intake might be a step that reduces the risk of respiratory diseases and promotes lung health.

### 4.9. Impact of Chronic Prenatal Exposure to the Food Additive Titanium Dioxide e171 on Respiratory Function in Newborn Mice

The study by Colnot et al. [16] analyzes the impact of prenatal exposure to titanium dioxide (E171), a common food additive, on newborn mice’s respiratory functions. E171 is a nanoparticulate form of titanium dioxide that is extensively used in food products as a whitening agent. This experiment was orally given to pregnant mice during gestation. This study aimed to evaluate the possible toxicity of E171, with a focus on its impact on the developing respiratory system of the offspring.

The results provided evidence concerning the impacts of E171 on changes in the infant mice’s respiratory function. These mice were marked by exhibiting an abnormally high respiratory frequency at postnatal day 3 and thus changed the initial control of respiration. In the first week of life, treated mice remained to have a highly elevated respiratory rate compared with controls: treated mice had an increase of 17% over that of the controls at postnatal day 7.

Electrophysiological analyses at postnatal days 5 to 6 indicated an increased excitability in the central neural circuits for respiration. Such results suggest that prenatal exposure to E171 interfered with the development of respiratory neural networks, thereby impairing the regulation of normal respiration.

The study raises concern about the nanoparticulate form of titanium dioxide because it may directly impede the development of necessary motor functions like breathing during critical periods of brain and neural development. Interference with these developments could result in direct respiratory problems and contribute to higher and long-term risks for developing respiratory control dysfunction.

The researchers highlight the risks of chronic exposure to food-grade titanium dioxide, especially for pregnant women and children. Since E171 is a common food additive across many pediatric diets, the findings show very serious safety concerns about its use in the early stages of development. The authors thus advocate for further research into fully understanding the long-term health impacts of E171 exposures—the effects on lung development and neurodevelopment.

All in all, the study, therefore, presents evidence of the possible dangers of titanium dioxide as a food additive, that is, the risk of damage due to its usage by deterring respiratory function in people vulnerable to such damage. It calls for further research into the safe use of E171 and other similar additives, which may well shape regulatory decisions on their future use in foodstuffs.

The current discussions on titanium dioxide’s safety and potential health effects led to controversial issues. The European Food Safety Authority (EFSA) in 2021 [17] re-evaluated titanium dioxide and concluded that it should be used as a food additive. France was the first country to ban its use as a food additive [18]. On the contrary, the United States Food and Drug Administration (FDA) considered it safe when used per the bylaws of good manufacturing practices [19]. Overall, the issues with titanium dioxide as a food additive depend on consumer awareness trends and regulatory transparency. As scientific understanding evolves, it remains crucial for regulatory agencies to assess food additives comprehensively, ensuring public health is prioritized [20,21].

## 5. Discussion

Tourmaa (1994) [22] notes that the consumption of food additives has increased significantly over the past decades. It is estimated that about 75% of the Western diet is now processed foods, and people consume an average of 8–10 lbs of food additives per year, and some even more. The consumption of these additives has been linked to several adverse health effects. A likely response to counter these effects would be the diminution of the intake of nonessential additives, which would limit the intake of nutrient-depleted foods and more likely compel the consumption of healthier foods [5].

Though the use of artificial food additives is regulated by international agencies such as the FDA (US) and EFSA (Europe), the prevalence of artificial food additives was influenced by local cuisines, consumer preference, and the food supply chain [23]. Various studies reveal artificial additives as potential health hazards, including hyperactivity in children, allergic complications, and potential carcinogenic effects [24]. It will always be good to replace artificial food additives with natural and organic food products [25]. Through community wellness programs, we need to educate the public on the safe use of natural food products [26]. Government agencies can play a major role in reducing the prevalence of artificial food additives [27]. This can include banning certain harmful additives and incentivizing manufacturers to use natural alternatives. Collaboration activities between industrialists and food scientists can foster innovation [28]. Legislative groups should eliminate artificial additives and support legislation aimed at improving food safety standards [29]. Promoting sustainable and organic farming practices can reduce the reliance on synthetic additives [30].

## 6. Future Directions

The following would constitute recommendations to resolve these issues [5]:

- Prohibition of all nonessential food additives, especially cosmetic additives such as food colorants.
- Regulatory agencies should ensure that only additives considered “generally recognized as safe” (GRAS) are allowed in food products.
- Foods containing additives with carcinogenic, mutagenic, or teratogenic properties should be clearly labeled with appropriate warnings.
- Additives should be prohibited in products intended for infants and young children.
- Non-GRAS additives should not exceed the established acceptable daily intake (ADI) limits.
- The amount of advertising on television of unhealthy junk food for children should be drastically reduced since children are very susceptible to advertisements that encourage bad eating habits.
- Food items that have no nutritional value or very low nutritional value should not be advertised.
- The food processing and manufacturing industries must be strictly followed by Good Manufacturing Practices (GMP) by the regulatory bodies.
- All foods, drinks, or medications that are at present exempt from declaring additives must be required to declare them in the future. This is especially necessary for medicines, as the current regulations on labeling do not require the declaration of pharmacological adjuvants. While adverse drug reactions are now recognized, synthetic excipients are no longer considered inert or nontoxic due to their growing use. In recurrent, unexplained symptoms, especially allergies and undisclosed excipients, should be taken into consideration.

## 7. Conclusions

This review recognizes the harmful effects of artificial food additives on lung health. The adverse effects of artificial food additives such as preservatives, synthetic colorants, and flavoring agents have been associated with asthma, COPD, and other respiratory disorders. All these additives can cause systemic inflammation, oxidative stress, and immune dysregulation, thereby exacerbating respiratory health. Sulfites and nitrites, preservatives, and Ponceau 4R, a colorant, cause allergic reactions and lung tissue damage. The gut-lung axis is an important factor in the amplification of these effects. Experimental studies on additives like titanium dioxide (E171) underscore the risks of prenatal exposure and occupational hazards from flavoring agents like diacetyl, further emphasizing additive safety concerns. Although artificial food additives are increasingly recognized and regulated as toxicants, researchers and policymakers may need to pay more attention to the possible toxicity of these food additives. Policymakers should ban these food additives that are more harmful to human health. In addition, they should also pay more attention to the food additives widely consumed by the human population. Funding research on long-term studies on these food additives should be encouraged. As an alternative to artificial additives, natural flavors and colors from fruits and vegetables, safe preservatives (vinegar, salt, etc.), and minimally processed ingredients can be used. In this way, we can save the human population from the health hazards of artificial food additives.

## Figures and Tables

**Table 1:** Artificial Food Additives and Toxicity on Lungs.

Food Additive	Mechanism of Action	Potential Effects on Lungs
Preservatives	Release reactive substances and trigger inflammatory responses	Increase inflammation and allergic reactions
Coloring Agents	Cause oxidative stress by generating free radicals	Damage lung tissue and impair function
Flavor Enhancers	Interact with immune response, leading to stress	Increase allergic reactions and reduce respiratory function
Artificial Sweeteners	Possible metabolic disruptions leading to stress response	Induce respiratory diseases
Emulsifiers	Alter gut microbiome and influence systemic inflammation	Increase the risk of inflammatory lung diseases

## Data Availability

This study is a review article; therefore, no new data were generated or analyzed.
